# Identification and classification of a new TRPM3 variant (γ subtype)

**DOI:** 10.1007/s12576-019-00677-6

**Published:** 2019-04-22

**Authors:** Kunitoshi Uchida, Naomi Fukuta, Jun Yamazaki, Makoto Tominaga

**Affiliations:** 1 Departments of Physiological Science and Molecular Biology and Morphological Biology, Fukuoka Dental College, Sawara-ku, Fukuoka, 814-0193 Japan; 2 Division of Cell Signaling, National Institute for Physiological Sciences, Okazaki Institute for Integrative Bioscience, National Institutes of Natural Sciences, Higashiyama 5-1, Myodaiji, Okazaki, Aichi 444-8787 Japan; 3 Department of Physiological Sciences, SOKENDAI (The Graduated University for Advanced Studies), Okazaki, Aichi 444-8585 Japan; 4 Thermal Biology Group, Exploratory Research Center on Life and Living Systems, National Institutes of Natural Sciences, Okazaki, Aichi 444-8787 Japan; 5 Institute for Environmental and Gender-Specific Medicine, Juntendo University, Chiba, 279-0021 Japan; 6 Present Address: Department of Veterinary Medicine, Nihon University College of Bioresource Sciences, Kanagawa, 252-0880 Japan

**Keywords:** TRPM3 channel, Variant, Ca^2+^-imaging, Oocyte recording, Thermosensitivity

## Abstract

TRPM3 is a non-selective cation channel that is activated by neural steroids such as pregnenolone sulfate, nifedipine, and clotrimazole. Despite the number of TRPM3 variants, few reports have described functional analyses of these different TRPM3 types. Here we identified a new TRPM variant from mouse dorsal root ganglion, termed TRPM3γ3. We classified TRPM3γ3 and another known variant (variant 6) into the γ subtype, and analyzed the TRPM3γ variants. mRNA expression of TRPM3γ was higher than that of TRPM3α variants in the mouse dorsal root ganglion. In Ca^2+^-imaging of HEK293 cells expressing either the TRPM3γ variants or TRPM3α2, increases in cytosolic Ca^2+^ concentrations ([Ca^2+^]_*i*_) induced by pregnenolone sulfate or nifedipine were smaller in cells expressing the TRPM3γ variants compared to those expressing TRPM3α2. On the other hand, co-expression of TRPM3γ variants had no effect on [Ca^2+^]_*i*_ increases induced by pregnenolone sulfate or nifedipine treatment of HEK293 cells expressing TRPM3α2. In *Xenopus* oocytes, small responses of TRPM3γ variants to chemical agonists compared to TRPM3α2 were also observed. Interestingly, *Xenopus* oocytes expressing TRPM3α2 displayed heat-evoked currents with clear thresholds of about 40 °C that were larger than those evoked in oocytes expressing TRPM3γ variants. Overall, these findings indicate that TRPM3γ variants have low channel activity compared to TRPM3α.

**Electronic supplementary material:**

The online version of this article (10.1007/s12576-019-00677-6) contains supplementary material, which is available to authorized users.

## Introduction

Most transient receptor potential (TRP) channels are non-selective cation channels. The name TRP is derived from the prototypical member in *Drosophila*, in which a mutation resulted in abnormally transient receptor potentials in response to continuous light exposure [1]. TRP channels are now divided into seven subfamilies: TRPC, TRPV, TRPM, TRPML, TRPN, TRPP, and TRPA, with six subfamilies (excluding TRPN) and 27 channels present in humans. TRP channels are expressed in many tissues and are involved in a wide variety of physiological functions, including detection of various physical and chemical stimuli in vision, taste, olfaction, hearing, touch, and thermosensation [2]. Recently, some channels were shown to have splicing variants that modulate channel and cell functions [3, 4]. Alternative splicing is a regulated process during gene expression that allows a single gene to encode multiple proteins having various functions. Among TRP channels, TRPM2, TRPM4, TRPM8, TRPV1, TRPC1, and TRPA1 are reported to have variants [5–14]. TRPM2 variants have different sensitivity to activators, wherein TRPM2-ΔN is insensitive to ADP ribose or H_2_O_2_ and TRPM2-ΔC is sensitive to H_2_O_2_ but not ADP ribose [7]. A truncated human TRPM8 variant is highly expressed in brain, liver, and testes, but not in other tissues where full-length TRPM8 is expressed [9]. A TRPV1 variant (TRPV1b) lacking 60 amino acids in the N-terminus is not activated by capsaicin or pH but retains heat sensitivity [10]. In addition, we previously reported that a short variant of mouse TRPA1 (TRPA1b) enhances the plasma membrane expression of wild-type TRPA1 (TRPA1a) and the expression of TRPA1b was increased during the development of neuropathic or inflammatory pain [8]. Since differences in functional modulation or expression pattern of TRP channel variants could affect channel and/or cellular functions to yield specific outcomes, a better understanding of TRP channel variant functions is needed to clarify the physiological and pathological roles of TRP channels.

TRPM3 was first identified as a store-operated ion channel [15] and is most prominently expressed in the kidney with lower expression levels in the central and peripheral nervous systems, testis, and retina [15, 16]. Several TRPM3 agonists, including the neural steroids pregnenolone sulfate, nifedipine, and clotrimazole, and TRPM3 inhibitors such as progesterone, flavanones, diclofenac, and mefenamic acid have been reported [17–22]. Physical stimuli such as hypo-osmolality also activate TRPM3 [23]. TRPM3 potentiates glutamatergic transmission at cerebellar Purkinje neurons [24] and is involved in insulin secretion [19], whereas TRPM3 gene polymorphisms may be associated with systemic sclerosis [25]. TRPM3 is activated by increases in temperature and TRPM3 expressed in sensory neurons is also involved in noxious heat sensation in vivo, as was shown in TRPM3KO mice [26]. Recently, it has been reported that G protein βγ inhibits TRPM3 activation [27, 28]. However, the physiological roles of TRPM3 channels remain unclear.

**Fig. 1:**
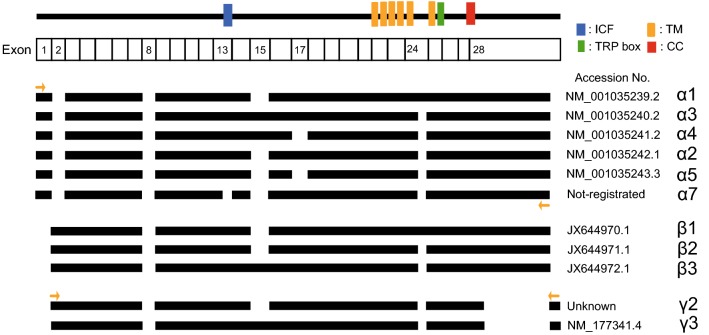
TRPM3 variant topology. The* uppermost line* indicates the membrane topology model of TRPM3.* Black bars* for each variant indicate coding regions.* Yellow allows* indicate primers for specific amplification of each variant. Primer sequences are shown in Table 1. Numbered exons indicate regions that are deleted in variants. *ICF* indispensable for channel function region, *TM* transmembrane domain, *CC* coiled-coil domain (color figure online)

The TRPM3 gene encodes more than ten variants [15, 29, 30]. These variants are mainly divided into two groups, α and β variants based on the variation of N-terminal (Fig. 1). While TRPM3αs lack exon 2, TRPM3βs lack exon 1 and a start codon of TRPM3βs is present in exon 2. Especially, most of the reports concerning these variants focused on the TRPM3αs [31]. Some reports showed functional differences among TRPM3α1, TRPM3α2, and TRPM3α7 [29, 31]. TRPM3α1 having inserted 12 amino acid residues in pore loop domain showed low permeability for Ca^2+^ and other divalent cations compared to TRPM3α2 that lacks 12 amino acid residues. TRPM3α7 that lacks ten amino acid residues within exon 13 was reported to be a non-functional channel and showed reduced plasma membrane expression. In addition, expression levels of variants lacking ten amino acid residues differ among tissues. On the other hand, TRPM3 variant 6 is very different from other variants in that the variant lacks 1169 bp sequence in exon 28 and splice 23 bp sequence in the rest of exon 28. However, functional analysis of the variant has not been reported.

In this study, we identified a new TRPM3 variant, which has sequence similarity to the previously reported TRPM3 variant 6 lacking a large part of exon 28. As such, we named the new variant γ3 and propose that variant 6 should be categorized into the same grouped as γ2 in the γ class. We measured the mRNA expression levels of TRPM3α and TRPM3γ variants in mouse dorsal root ganglion, and analyzed the functions of TRPM3γ2 and γ3 variants relative to TRPM3α2. In addition, we analyzed the temperature sensitivity of these TRPM3 variants in *Xenopus* oocytes.

## Materials and methods

### Animals

Male C57BL/6 mice (4–6 weeks, SLC, Shizuoka, Japan) were housed in a controlled environment (12-h light/dark cycle, room temperature 22–24 °C, 50–60% relative humidity) with free access to food and water. All procedures involving the care and use of animals were approved by The Institutional Animal Care and Use Committee of the National Institutes of Natural Sciences and performed in accordance with the Guide for the Care and Use of Laboratory Animals (National Institutes of Health Publication Number 85-23, Revised 1985).

### Identification of TRPM3 variants

Dorsal root ganglion was isolated from male C57BL/6 mice under deep anesthesia by sevoflurane, and total RNA was purified using an RNeasy Mini Kit (QIAGEN, Hilden, German). cDNA was synthesized from 1 μg of total RNA (Superscript III first-strand synthesis system for RT-PCR; Invitrogen, Carlsbad, CA, USA). Two-step nested PCR was performed using Phusion High-Fidelity DNA polymerase (New England Biolabs, Ipswich, MA, USA) in an iCycler (Bio-Rad, Hercules, CA, USA) with specific primer sets for each TRPM3 variant (Table 1). The primer pairs were designed in 5′ UTR and 3′UTR of each variant for the first amplification, and designed at either end of CDS for the second amplification. Following cycle protocols were applied: For the first amplification, 35 cycles were performed each with incubation at 98 °C for 10 s, followed by 67 °C for 30 s, 72 °C for 160 s. For the second amplification, 35 cycles were performed each with incubation at 98 °C for 10 s, followed by 67 °C for 30 s, 72 °C for 160 s. PCR products were purified using a NucleoSpin Gel and PCR Clean-up kit (Macherey–Nagel, Duren, Germany) and were subcloned into pcDNA3.1. The entire sequences of each TRPM3 variant were confirmed by sequencing (BigDye Terminator V3.1 and ABI PRISM 3130xl analyzer, Applied Biosystems Inc., Carlsbad, CA, USA).

**Table 1:** Primer sets for cloning of TRPM3α and γ variants

	Forward(5′–3′)	Reverse(5′–3′)
TRPM3α for 1st amplification	GAGAGCTGAGCGCAGGCTG	TCCTGCAACACACGGTAAGCC
TRPM3α for 2nd amplification of target cDNA	ATGGGCAAGAAGTGGAGGGATG	TTAGTTGTGCTTGCTTTCAAAGC
TRPM3γ for 1st amplification	CCAGGAAGCCTCTGCCCTAA	AACTGCTTGCTGCCGGCTTA
TRPM3γ for 2nd amplification of target cDNA	ATGCCAGGGCCGTGGGGGAC	TTATACTGAATAAAAAGGATGTTCTGC
TRPM3α+β+γ for real-time PCR	CGGCCATCATGGCCTGCTAC	GGCAGAACCGGCCTCTCG
TRPM3α for real-time PCR	CTCTGACCGCGAGGACAGCA	GCAGTCCCGAGCGCTTGGTG
TRPM3γ for real-time PCR	CTTATACTGAATAAAAAGGATGTTCTGCAG	CTCTGACCGCGAGGACAGCA
β-actin for real-time PCR	TGTTACCAACTGGGACGACA	AAGGAAGGCTGGAAAAGAGC

### Quantitative real-time RT-PCR

**Fig. 2:**
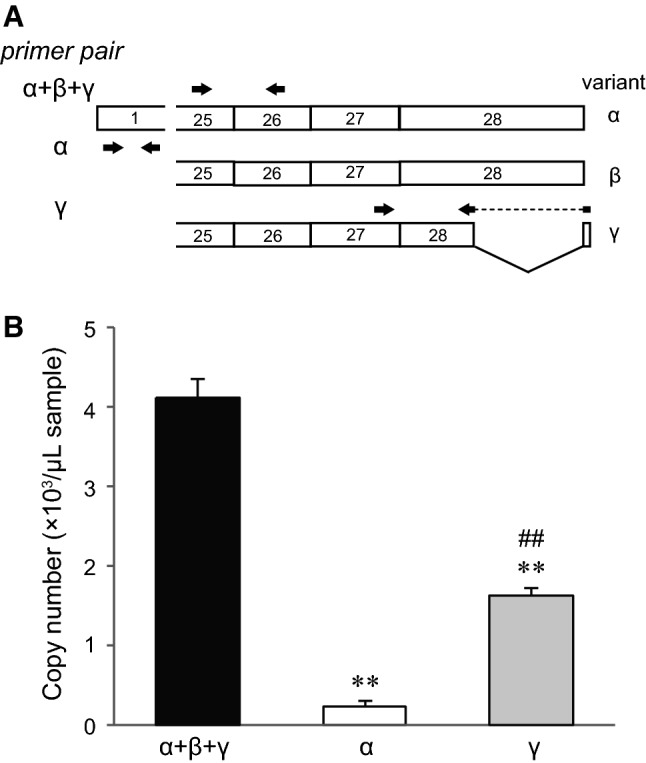
Expression levels of TRPM3α and TRPM3γ mRNA in mouse dorsal root ganglion. **a** Design of primer sets for quantifying the TRPM3 variants. Different primer pairs and their location relative to the TRPM3 mRNA are shown as* arrows*. Primer sequences are shown in Table 1. **b** mRNA expressions of TRPM3 variants (α+β+γ, α, and γ) in mouse dorsal root ganglion by quantitative real-time RT-PCR analysis.* Y-axis*: Copy number of mRNAs per 1 μl mRNA sample. Each* bar* represents the mean + SEM of six mice. Statistical significance was assessed using ANOVA followed by a two-tailed multiple *t* test with Bonferroni correction. **P < 0.01 versus TRPM3α+β+γ, ^##^*P* < 0.01 versus TRPM3α

Total RNA isolation and cDNA synthesis from mouse dorsal root ganglion were performed as described above. Real-time reverse transcription-polymerase chain reactions (RT-PCR) were performed using specific primers (Table 1, Fig. 2, and also described in the Results section) and Power SYBR Green PCR Master Mix (Applied Biosystems). Diluted TRPM3α2/pcDNA3.1 or TRPM3γ2/pcDNA3.1 plasmid solution was used for the calibration curve. The duplex real-time RT-PCR was performed with the PlusOne Real-Time PCR system (Applied Biosystems). Transcriptional levels of TRPM3α+β+γ, TRPM3α and TRPM3γ variants were determined by the calculation of copy numbers. To confirm the RNA isolation and cDNA synthesis, amplification of β-actin was performed and the mean value of Cq was 20.8 ± 0.2 (mean ± SD, *n* = 6).

### Ca^2+^-imaging

Human embryonic kidney-derived 293T (HEK293T) cells were maintained in DMEM (WAKO Pure Chemical Industries, Ltd., Osaka, Japan) containing 10% FBS (Biowest SAS, Caille, France), 100 units/ml penicillin (Invitrogen, Carlsbad, CA, USA), 100 mg/ml streptomycin (Invitrogen), and 2 mM l-glutamine (GlutaMAX, Invitrogen) at 37 °C in 5% CO_2_. Plasmid DNAs (0.2 μg) and 0.03 μg pCMV-DsRed-expression cDNAs were transfected into HEK293T cells using Effectene Reagent (QIAGEN). One day after transfection, HEK293T cells on coverslips were mounted in an open chamber and superfused with a standard bath solution (140 mM NaCl, 5 mM KCl, 2 mM MgCl_2_, 2 mM CaCl_2_, 10 mM HEPES, 10 mM glucose, pH 7.4). All chemicals were dissolved in the standard bath solution, and a heated bath solution was applied for heat stimulation (~ 42 °C). Cytosolic-free Ca^2+^ concentrations ([Ca^2+^]_*i*_) in HEK293T cells were measured by dual-wavelength fura-2 microfluorometry (excitation at 340/380 nm and emission at 510 nm, Molecular Probes, Invitrogen) and a digital CMOS camera (Andor Zyla 5.5, Andor Technology Ltd, Belfast, UK). All experiments were performed at room temperature except for heat stimulation. The ratio image was calculated and acquired using Andor iQ2 (Andor Technology Ltd) or ImageJ (NIH). Ratio values were normalized with respect to the peak response to 5 μM ionomycin (Dojindo Laboratories, Kumamoto, Japan).

### Expression of EGFP-tagged TRPM3 variants in HEK293T cells

EGFP-tagged TRPM3α2, TRPM3α3, TRPM3γ2, or TRPM3γ3 plasmid (1.2 μg) was transfected into HEK293T cells using Lipofectamine and Plus Reagent (Thermo Fisher Scientific Inc.) in OPTI-MEM (Thermo Fisher Scientific Inc.). Transfected-HEK293T cells were incubated for 3 h at 37 °C in a 5% CO_2_ atmosphere. After incubation, the cells were reseeded on glass-bottom dishes in DMEM and further incubated under the same conditions. After an additional day of incubation, images of HEK293T cells were acquired using a confocal microscopy (FV1200, Olympus Corporation, Tokyo, Japan) and FV10-ASW 4.2 software (Olympus Corporation).

### Oocyte recording

The TRPM3 variants were heterologously expressed in oocytes of the African clawed frog *Xenopus laevis*, and the two-electrode voltage-clamp method was used for current recordings. A total of 2.5 ng cRNA was injected into defolliculated oocytes and current recordings were performed 4–5 days after injection. The membrane potential of oocytes was clamped at − 60 mV. To generate the current–voltage (*I*–*V*) curve, voltage ramp-pulses from − 80 to + 80 mV (500 ms) were applied every 5 s. ND96 solution containing 93.5 mM NaCl, 2 mM KCl, 1.8 mM CaCl_2_, 2 mM MgCl_2_, 5 mM HEPES, pH 7.5 (with NaOH) was perfused and all chemicals were dissolved in the bath solution. Chemicals were applied for 1 min except for those currents that peaked within 1 min. For temperature stimulation, heated or chilled ND96 solutions were applied by perfusion. All experiments were performed at room temperature except for those involving cold and heat stimulation. Data were sampled at 5 kHz and filtered at 1 kHz for analysis using an OC-725C oocyte clamp (Warner Instruments) with pCLAMP10 software (Axon Instruments, CA, USA). *Xenopus* oocytes injected with a distilled water (DW) were used for control experiments.

### Statistical analysis

Data are expressed as mean ± SEM. Statistical analysis was performed by Student’s *t* test or one-way analysis of variance (ANOVA), followed by a two-tailed multiple *t* test with Bonferroni correction. *P* values < 0.05 were considered significant.

## Results

We attempted to clone TRPM3 variants from mouse dorsal root ganglion mRNA using a nested PCR method and sequence specific primers (Table 1, some were used in previous cloning studies). In mouse dorsal root ganglion, we found four TRPM3 variants; α2, α3, variant 6 and an unknown variant (Fig. 1). Although the pattern of N- and C-termini sequence was shared between variant 6 and the unknown variant, the characteristics of these two differed from those for the α or β variants. Accordingly, we classified variant 6 and the unknown variant as γ variants termed TRPM3γ2 and TRPM3γ3 for the unknown variant and variant 6, respectively. This naming convention mirrors that for TRPM3α2 and TRPM3α3 because of the absence and presence, respectively, of exon 15.

First, to elucidate the expression level of TRPM3α and TRPM3γ variants in sensory neurons, we measured their mRNA expression levels in mouse dorsal root ganglion by real-time RT-PCR. We constructed the primer sets in exon 1 for measuring the TRPM3α and in exon 27 and 28 for measuring the TRPM3γ variant (Fig. 2a). In addition, we constructed the primer sets in exon 25 and 26 for measuring the total mRNA expression level of TRPM3α, TRPM3β and TRPM3γ, except TRPM3β15 and 16, which are deleted from exon 20–28 containing transmembrane domain and C-terminal region [30] (Fig. 2a). As shown in Fig. 2b, we confirmed that total TRPM3 mRNA expression level was higher than TRPM3α and TRPM3γ (Fig. 2b). TRPM3γ mRNA level was much higher than that of TRPM3α (Fig. 2b).

**Fig. 3:**
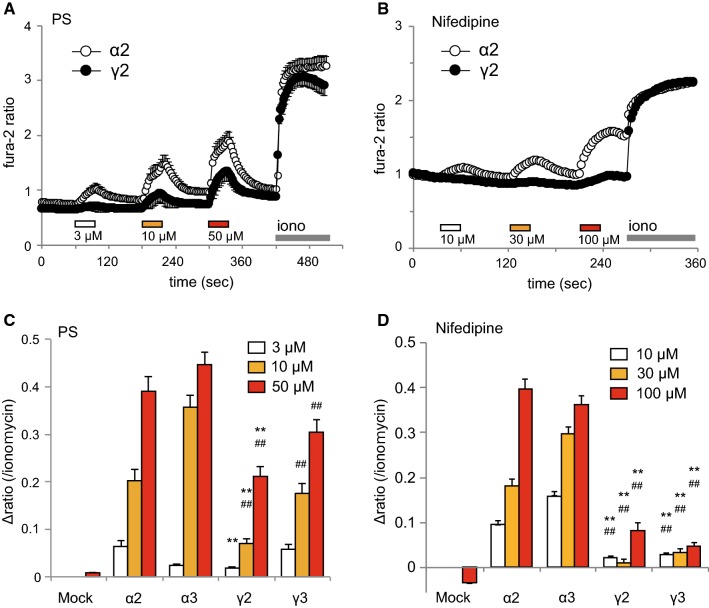
[Ca^2+^]_*i*_ increases induced by pregnenolone sulfate or nifedipine treatment of HEK293T cells expressing mouse TRPM3γ or TRPM3α variants. **a**, **b** Average changes in [Ca^2+^]_*i*_ induced by pregnenolone sulfate (PS, **a**) or nifedipine (**b**) treatment of HEK293T cells expressing TRPM3α2 or TRPM3γ2. Cell viability was confirmed by application of 5 μM ionomycin (Iono). The* y-axis* shows the fura-2 ratio of 340 nm/380 nm. Each* symbol* represents mean ± SEM of 51–108 cells. **c**, **d** Effect of 3, 10, or 50 μM PS (**c**) and 10, 30, or 100 μM nifedipine (**d**) on HEK293T cells expressing mouse TRPM3α2, TRPM3α3, TRPM3γ2, or TRPM3γ3. Mock shows results for vector-transfected HEK293T cells.* Y-axis*: the Δ ratio normalized to ionomycin responses. Each column represents the mean + SEM of 80–280 cells. Statistical significance was assessed using ANOVA followed by a two-tailed multiple *t* test with Bonferroni correction. **P < 0.01 versus TRPM3α2, ^##^*P* < 0.01 versus TRPM3α3

**Fig. 4:**
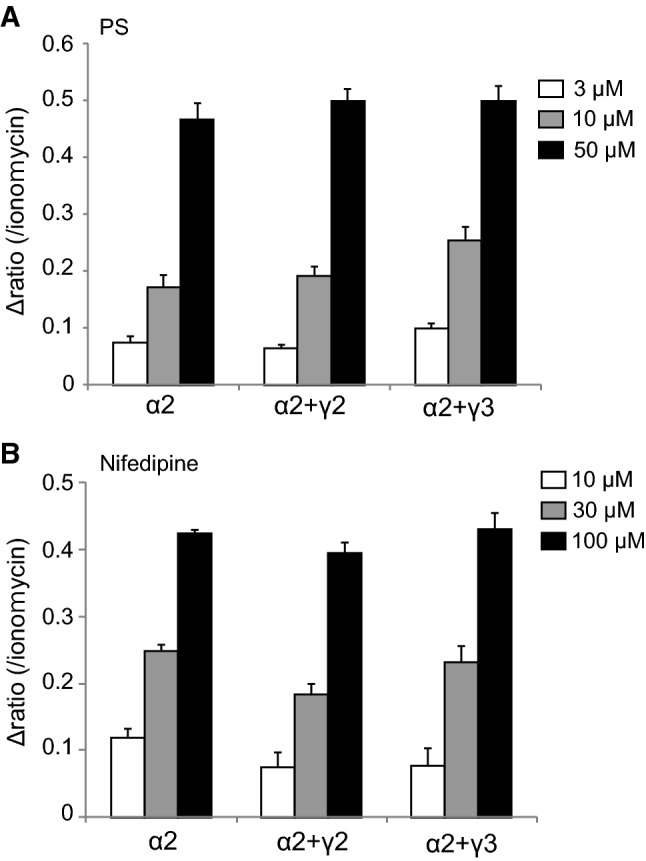
Effects of TRPM3γ2 or TRPM3γ3 co-expression with TRPM3α2 on [Ca^2+^]_*i*_ changes induced by pregnenolone sulfate or nifedipine. [Ca^2+^]_*i*_ increases induced by pregnenolone sulfate (PS, **a**) or nifedipine (**b**) treatment of HEK293T cells expressing mouse TRPM3α2 without or with TRPM3γ2 or TRPM3γ3.* Y-axis*: the Δ ratio normalized to ionomycin responses. Each column represents the mean + SEM of 95–171 cells

To characterize the functions of these TRPM3γ variants, we first compared the responses to pregnenolone sulfate (PS) or nifedipine in HEK293 cells expressing TRPM3α2, TRPM3α3, TRPM3γ2, or TRPM3γ3 using Ca^2+^-imaging. Increases in [Ca^2+^]_*i*_ induced by treatment with 3, 10, or 50 μM PS were small for HEK293 cells expressing TRPM3γ2 compared to cells expressing TRPM3α2 (Fig. 3a). Treatment with 10, 30, or 100 μM nifedipine resulted in smaller [Ca^2+^]_*i*_ increases in HEK293 cells expressing TRPM3γ2 relative to those expressing TRPM3α2 (Fig. 3b). Overall, treatment of HEK293 cells expressing TRPM3γ2 or TRPM3γ3 with PS or nifedipine produced significantly smaller [Ca^2+^]_*i*_ increases than those in cells expressing TRPM3α2 or α3 (Fig. 3c, d). On the other hand, when we examined the expression of TRPM3 variants in HEK293T cells using EGFP-tagged TRPM3α2, TRPM3α3, TRPM3γ2, or TRPM3γ3, there was no clear difference in EGFP signals among the variants (Supplemental Figure 1). We next examined the possible interaction between TRPM3α and TRPM3γ variants using TRPM3α2 co-expressed in HEK293 cells with either TRPM3γ2 or TRPM3γ3. Co-expression of TRPM3α2 and TRPM3γ variants did not affect PS- or nifedipine-induced [Ca^2+^]_*i*_ increases (Fig. 4), suggesting that these channel proteins do not have functional interaction.

**Fig. 5:**
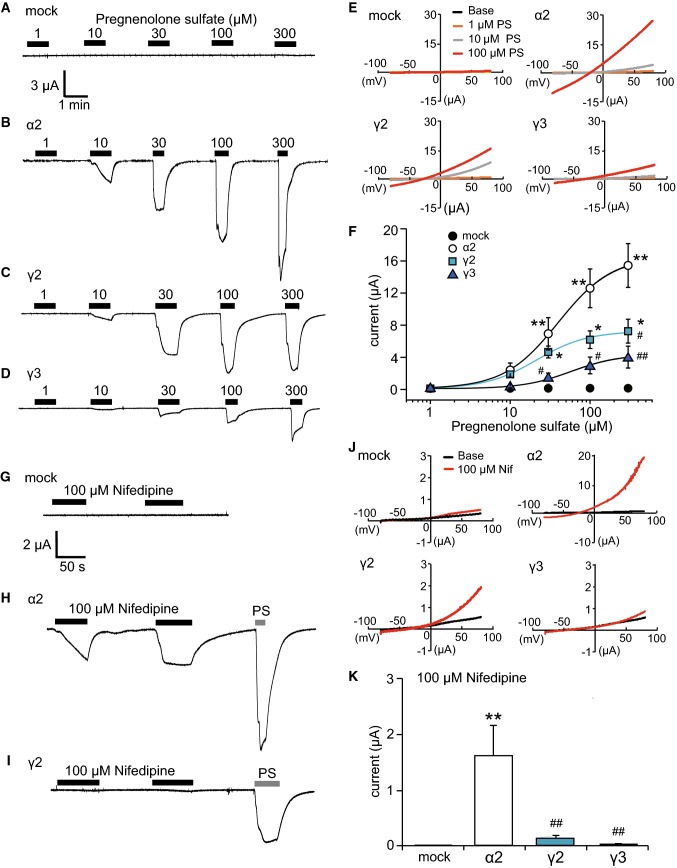
Currents activated by pregnenolone sulfate or nifedipine in *Xenopus* oocytes expressing mouse TRPM3α2, TRPM3γ2, or TRPM3γ3. **a**–**d** Representative traces of endogenous (mock, **a**), TRPM3α2 (**b**), TRPM3γ2 (**c**), or TRPM3γ3 (**d**) currents activated by treatment of *Xenopus* oocytes with 1–300 μM pregnenolone sulfate. The membrane potential was held at − 60 mV. **e** Representative current–voltage curves of the currents by treatment of distilled water-injected *Xenopus* oocytes (mock) or *Xenopus* oocytes expressing TRPM3α2, TRPM3γ2, or TRPM3γ2 with 1, 10, and 100 μM pregnenolone sulfate (PS). **f** Dose–response profiles of currents in *Xenopus* oocytes expressing TRPM3α2, TRPM3γ2, or TRPM3γ3 activated by pregnenolone sulfate. Mock indicates the currents by pregnenolone sulfate in distilled water-injected *Xenopus* oocytes. Hill coefficients were 1.3 ± 0.1 (TRPM3α2), 1.4 ± 0.2 (TRPM3γ2) and 1.5 ± 0.2 (TRPM3γ3). Each symbol represents the mean ± SEM of 9–11 oocytes. Statistical significance was assessed using ANOVA followed by a two-tailed multiple *t* test with Bonferroni correction. *P < 0.05, **P < 0.01, versus DW, ^#^*P* < 0.05, ^##^*P* < 0.01, versus TRPM3α2. **g**–**i** Representative traces of endogenous (mock, **g**), TRPM3α2 (**h**) or TRPM3γ2 (**i**) currents activated by 100 μM nifedipine in *Xenopus* oocytes. The membrane potential was held at − 60 mV. Pregnenolone sulfate (PS, 100 μM) was applied in the end. **j** Representative current–voltage curves of the currents by 100 μM nifedipine in distilled water-injected *Xenopus* oocytes (mock) or *Xenopus* oocytes expressing TRPM3α2, TRPM3γ2, or TRPM3γ3 with 100 μM nifedipine (Nif). **k** Comparison of peak nifedipine (100 μM)-evoked currents in distilled water-injected *Xenopus* oocytes (mock), and *Xenopus* oocytes expressing TRPM3α2, TRPM3γ2, or TRPM3γ3. Each column represents the mean ± SEM of 5–10 oocytes. Statistical significance was assessed using ANOVA followed by a two-tailed multiple *t*-test with Bonferroni correction. **P < 0.01, versus DW, ^##^*P* < 0.01, versus TRPM3α2

To evaluate the function of each TRPM3 variant, we next performed an electrophysiological study. We first confirmed that 1–300 μM PS did not cause any activating current in DW-injected *Xenopus* oocytes (Fig. 5a). A comparison of representative traces of dose-dependent currents at − 60 mV induced by 1–300 μM PS in *Xenopus* oocytes expressing TRPM3α2, TRPM3γ2, or TRPM3γ3 showed that the currents were small for the γ variants relative to oocytes expressing TRPM3α2 (Fig. 5b–d), which is consistent with the Ca^2+^-imaging results (Fig. 3). Although the 300 μM PS-evoked currents did not appear to be saturated in oocytes expressing TRPM3α2, due to low solubility we could not use higher PS concentrations. Next, we evaluated the current–voltage relationship of TRPM3α2, TRPM3γ2, and TRPM3γ3. *I*–*V* curves from − 80 to + 80 mV in the presence of 10 or 100 μM PS showed outward rectification, and did not differ among the variants (Fig. 5e). We then examined dose–response profiles of these variants. The EC_50_ values were 39.8 ± 1.7, 21.5 ± 3.0 and 57.4 ± 7.3 μM for TRPM3α2, TRPM3γ2, and TRPM3γ3, respectively (Fig. 5f), indicating that the potency of PS is similar among the variants. On the other hand, PS-evoked current sizes were significantly smaller in TRPM3γ2 and TRPM3γ3 compared with TRPM3α2 (Fig. 5f), indicating a difference in efficacy. Meanwhile, in *Xenopus* oocytes expressing TRPM3α2, currents activated by 100 μM nifedipine (the highest concentration that could be tested due to solubility) at − 60 mV were much smaller than those activated by PS. Moreover, almost no nifedipine-evoked currents at − 60 mV were observed in DW-injected *Xenopus* oocytes and *Xenopus* oocytes expressing TRPM3γ2 or TRPM3γ3 (Fig. 5g, i). In the presence of 100 μM nifedipine, *I*–*V* curves from − 80 to + 80 mV showed outward rectification, and were similar among the variants (Fig. 5j). Nifedipine-evoked current sizes were significantly smaller in TRPM3γ2 and TRPM3γ3 compared with TRPM3α2 (Fig. 5k), which is similar to that of PS. Because the washout of nifedipine-evoked currents in *Xenopus* oocytes expressing TRPM3α3 was very slow (Supplemental Figure 2A and B), the nifedipine data were not analyzed further.

**Fig. 6:**
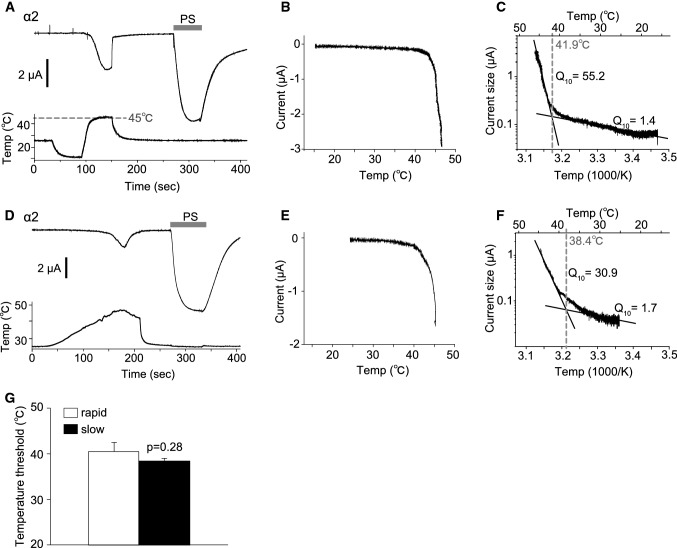
Temperature-activated currents in *Xenopus* oocytes expressing mouse TRPM3α2. **a**, **d** Representative traces of TRPM3α2 currents activated by rapid (**a**) or slow (**d**) temperature changes up to 45 °C in *Xenopus* oocytes. Pregnenolone sulfate (PS, 100 μM) was applied after the temperature stimulus and the membrane potential was held at − 60 mV. **b**, **e** Temperature–current profiles from the traces in **a** and **d**, respectively. The *x*- and *y*-axes show temperature (°C) and current (μA), respectively. **c**, **f** Arrhenius plots from the traces in **a** and **d**, respectively. The lower and upper *x*-axes show 1000/temperature (K) and temperature (°C), respectively, whereas the *y*-axis shows the common logarithmic plot of current size. *Q*_10_ values were calculated from the approximate lines shown in* black*. The intersection of the two linear-fitted lines was defined as a temperature threshold as shown by the* dashed line*. **g** Comparison of the temperature thresholds for rapid and slow heat-evoked TRPM3α2 activation. Each* column* represents the mean ± SEM of 6–8 oocytes. Statistical significance was assessed using Student’s *t* test

**Fig. 7:**
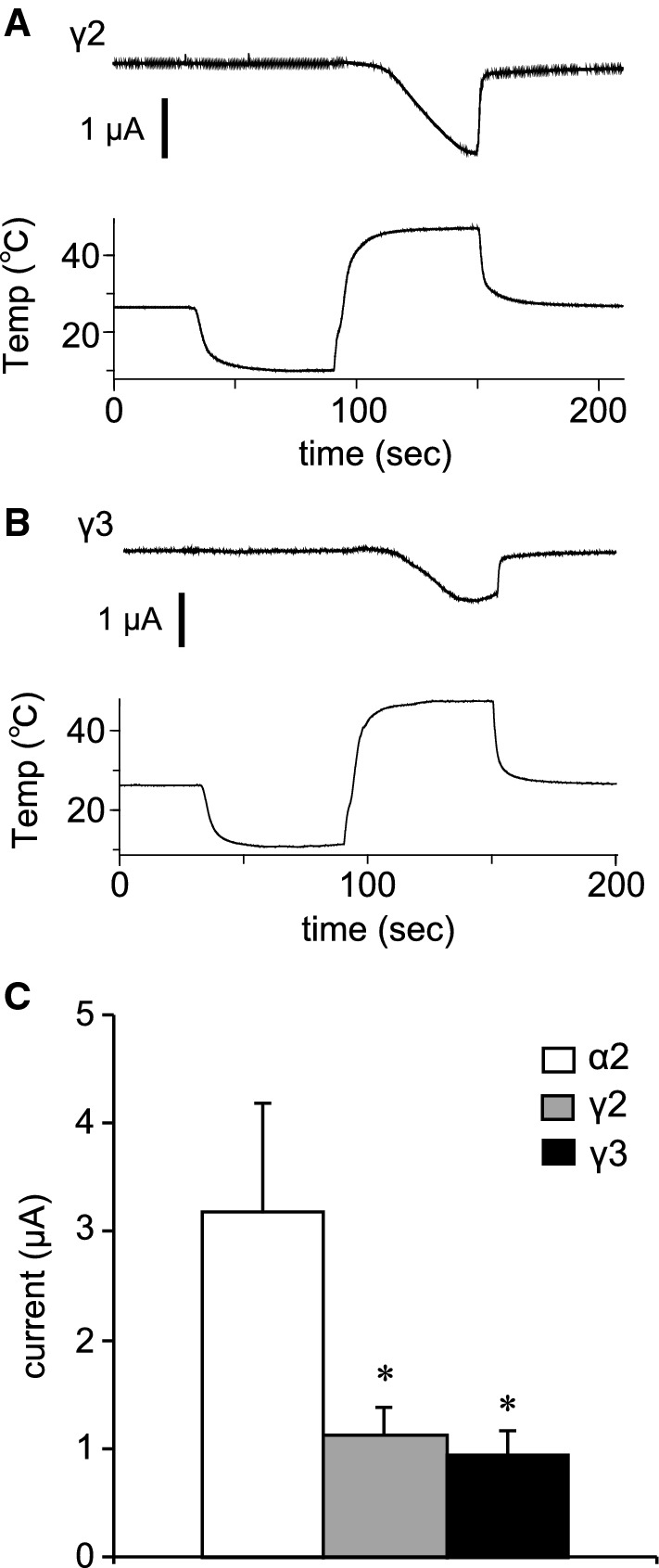
Temperature-activated currents in *Xenopus* oocytes expressing mouse TRPM3γ2 or TRPM3γ3. **a**, **b** Representative traces of TRPM3γ2 (**a**) or TRPM3γ3 (**b**) currents activated by rapid temperature changes up to 45 °C in *Xenopus* oocytes. **c** Comparison of sizes of heat-evoked currents in *Xenopus* oocytes expressing TRPM3α2, TRPM3γ2, or TRPM3γ3. Each* column* represents the mean ± SEM of 7–8 oocytes. Statistical significance was assessed using ANOVA followed by a two-tailed multiple *t* test with Bonferroni correction. *P < 0.05 versus TRPM3α2

We also examined temperature-dependent current responses of TRPM3 variants. Although a reduction in temperature induced no current activation, an acute temperature increase (~ 46 °C) activated currents in *Xenopus* oocytes expressing TRPM3α2 (Fig. 6a), which is consistent with an earlier report [26]. These acute-heat-activated currents were not observed when the temperature was increased up to 40 °C (Supplemental Figure 3A), and control *Xenopus* oocytes injected with distilled water showed only very small currents upon heating to 46 °C (Supplemental Figure 3B). Temperature–current response profiles showed significant currents above 40 °C, consistent with the lack of currents below 40 °C (Fig. 6b). Arrhenius plots generated to obtain *Q*_10_ values and temperature thresholds for channel activation showed *Q*_10_ values before and after activation of 1.4 and 55.2, respectively, and the temperature threshold was 41.9 °C in that particular oocyte (Fig. 6c). Heat-activated TRPM3α2 currents were also observed when the temperature was increased slowly (Fig. 6d). Analysis of temperature response profiles (Fig. 6b, c) indicated a temperature threshold of 38.4 °C and *Q*_10_ values before and after activation of 1.7 and 30.9, respectively (Fig. 6e, f). Although the temperature thresholds were somewhat low when the temperature was increased slowly, there were no statistically significant differences in the threshold between slow and rapid heating (rapid: 40.51 ± 1.95 °C and slow: 38.41 ± 0.67 °C, Fig. 6g). The heat-activated currents seen in *Xenopus* oocytes expressing TRPM3γ2 and TRPM3γ3 were significantly smaller than those of TRPM3α2 (Fig. 7), which is similar to the difference in current responses caused by chemical agonists.

## Discussion

In this study, we identified two new TRPM3 variants that we classified into a new subtype, γ, according to differences in the N- and C-termini. Analysis of the functions of these two new variants, TRPM3γ2 and γ3, showed that these channels were activated to a low degree by both chemical ligands and heat stimulation relative to the TRPM3α variant. Electrophysiological analysis in *Xenopus* oocytes showed that the TRPM3α2 variant had temperature thresholds around 40 °C.

TRPM3 has many variant types, and more than ten different variants have been reported [30]. These variants can be divided into two main groups, α and β, depending on the presence of deletions of exon 1, 2 or 28, which contain the start codon or stop codon [31]. However, the functional analyses of TRPM3 variants are largely limited to the α variant. The TRPM3α1 variant has a large extracellular pore region and different divalent cation permeability relative to TRPM3α2 [29]. TRPM3α7 lacks the ICF (indispensable for channel function) region in exon 13 (TRPM3ΔICF, indicated by the blue square in Fig. 1), and this TRPM3ΔICF variant has reduced interaction with other TRPM3 isoforms, as well as reduced localization to cell membranes [31]. Our finding that TRPM3γ2 and γ3 had low activation relative to the TRPM3α2 variant in response to both chemical ligands and temperature in HEK293T and *Xenopus* oocytes (Figs. 3 and 5) could be due to several possibilities: (1) lower protein expression; (2) impaired tetramerization; (3) impaired translocation to the plasma membrane; or (4) lower channel activity of the variants. Some reports demonstrated that coiled–coil region in C-terminus of TRPM8 and other TRPM channels are involved in tetramerization [32, 33]. The coiled-coil region in C-terminus is conserved in TRPM3γ variants, ruling out the possibility of impaired tetramerization. We found that the potency of the TRPM3γ in response to PS treatment was much lower than that of the TRPM3α2 variant, although the dose-dependency of PS and nifedipine responses could not be pursued due to the low solubility of those compounds. We propose that the TRPM3γ variants could have both reduced protein expression (including membrane expression) and/or impaired channel activity. We confirmed that the transfected HEK293T cells indeed express TRPM3 variant proteins (Supplemental Figure 1), but further analysis is needed to quantify difference in protein expression levels among the variants. In this study, we found that a comparison of the TRPM3γ2 and γ3 sequences showed differences in exon 15, which may be the basis for the lower responses to PS than those seen for TRPM3α variants. On the other hand, TRPM3α3 showed high activity to chemical ligands compared to TRPM3α2 (Fig. 5 and Supplemental Figure 2). Although, a previous study suggested that splicing within exons 8, 15, and 17 did not affect TRPM3-mediated [Ca^2+^]_*i*_ increases stimulated by 30 μM PS or increase protein expression levels [31], our data suggested that exon 15 modulates channel activity to some extent. In addition, co-expression of TRPM3γ variants with TRPM3α2 in HEK293 cells did not affect [Ca^2+^]_*i*_ increases stimulated by ligands (Fig. 4), which is in contrast to an earlier report showing that co-expression of TRPM3ΔICF variant with other TRPM3α variants reduced the number of channels, and impaired TRPM3-mediated [Ca^2+^]_*i*_ increases [31]. Thus, TRPM3γ variants could not modulate the activity of TRPM3α variants stimulated by PS or nifedipine. Nevertheless, to clarify the functional significances of TRPM3γ variants, further analysis is necessary.

An exploration of expression profiles for TRPM3 variants is important to determine whether the variants affect the activity of other variants. However, detection of the specific variants at the protein and mRNA levels by immunohistochemistry and in situ hybridization at a single cell level, respectively, is challenging due to the similarity in DNA sequence. In this study, while it was from tissue but not from cells, we succeeded in comparing the mRNA expression levels between TRPM3α and TRPM3γ variants by real-time RT-PCR with specific primers (Fig. 2). In addition, the mRNA expression level of TRPM3γ variants was significantly higher than TRPM3α variants in mouse dorsal root ganglion, suggesting that the mRNA expression level of TRPM3α variants is lower than that of TRPM3β variants (Fig. 2). Therefore, it is important to analyze not only TRPM3α but also TRPM3β and TRPM3γ variants in order to understand the physiological significances of TRPM3.

We first observed a clear temperature threshold for heat-evoked activation of TRPM3 variants in *Xenopus* oocyte recordings, which showed temperature-dependent activation only for TRPM3α2 that had a temperature threshold of around 40 °C (Fig. 6). A 2011 study by Vriens et al. demonstrated temperature-dependent activation of TRPM2α2 and also showed that TRPM3KO mice had impaired avoidance behaviors from noxious heat [26]. Moreover, they found that temperature-dependent avoidance behavior at or above 45 °C differed depending on the measurement method [26]. In particular, the temperature threshold in vivo was slightly higher than that seen in *Xenopus* oocyte recordings. A similar difference between the in vitro and in vivo temperature threshold, > 40 °C and ≥ 52.5 °C, respectively, was also reported for TRPV1KO mice [34]. The Vriens et al. [26] study also revealed that both HEK293 cells expressing TRPM3α2 and dorsal root ganglion neurons showed TRPM3-dependent activation in response to increased temperature. We recently reported that TRPM3α2 protein reconstituted into lipid bilayers has diminished temperature dependency [35]. However, in the presence of PI(4, 5)*P*_2_, TRPM3α2 exhibited *Q*_10_ of 5.3, which is relatively close to that observed in cells (*Q*_10_= 7.2) [26]. On the other hand, TRPM3α2 exhibited *Q*_10_≥ 30 in *Xenopus* oocytes. These temperature-dependent properties of TRPM3 thus differed from the other thermo-TRPs, TRPV1 and TRPM8. In planar lipid bilayer experiments, the temperature dependence of TRPM8 is *Q*_10_= 40, whereas that for TRPV1 is *Q*_10_= 18 [36, 37], and in heterologous expression experiments the *Q*_10_ for both TRPV1 and TRPM8 is more than 10 [38, 39], indicating that in some experimental settings the differences between these TRP channels are not large. As we concluded previously, some molecules, including PI(4,5)*P*_2_, could be necessary for temperature-dependent TRPM3 activation [35], while some of these candidate molecules could also exist in *Xenopus* oocytes. Although the mechanisms of temperature-dependent activation of TRPM3 await further characterization, this channel family could act as sensors of temperatures above 40 °C.

## Electronic supplementary material

Below is the link to the electronic supplementary material.

Supplementary Figure 1. Confirmation of TRPM3 variant expression in HEK293T cells after transfection with TRPM3α2, TRPM3α3, TRPM3γ2, or TRPM3γ3 plasmids. Left and middle panels show fluorescence (left) and bright-field (middle) images of HEK293T cells expressing EGFP-tagged TRPM3α2, TRPM3α3, TRPM3γ2, or TRPM3γ3. Scale bar: 70 µm. Right panels show magnified fluorescent (upper) and bright-field (lower) images of a single HEK293T cell. Scale bar: 20 µm. (PDF 5262 kb)

Supplementary Figure 2. (**A**) Representative trace of TRPM3α3 currents activated by pregnenolone sulfate (1 to 300 µM). (**B**) Representative trace of TRPM3α3 currents activated by nifedipine (100 µM). Pregnenolone sulfate (PS, 100 µM) was applied after nifedipine. (PDF 841 kb)

Supplementary Figure 3. (**A**) Representative trace of TRPM3α2 currents activated by rapid temperature changes up to 40 °C in *Xenopus* oocytes. Pregnenolone sulfate (PS, 100 µM) was used to confirm TRPM3α2 expression. (**B**) Representative trace of currents induced by rapid temperature changes up to 46 °C in water-injected *Xenopus* oocytes. (PDF 521 kb)
