# Supplementary figures and images for: Identification and classification of a new TRPM3 variant (γ subtype)
Source: J Physiol Sci. 2019 Apr 22;69(4):623–34. doi: 10.1007/s12576-019-00677-6 (PMC6583685; doi:10.1007/s12576-019-00677-6)

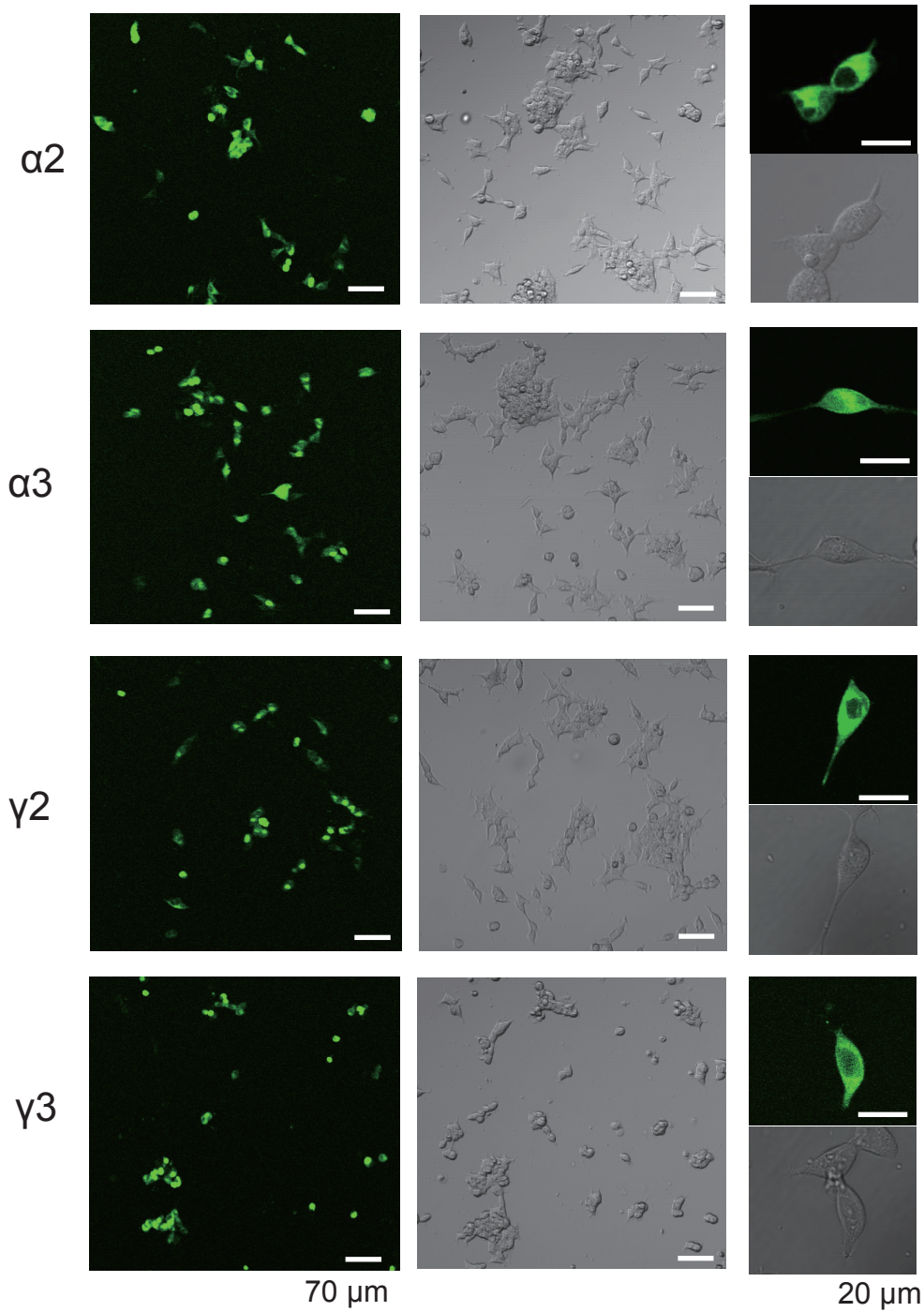

Supplement: Supplementary file 1 — Supplementary Figure 1. Confirmation of TRPM3 variant expression in HEK293T cells after transfection with TRPM3α2, TRPM3α3, TRPM3γ2, or TRPM3γ3 plasmids. Left and middle panels show fluorescence (left) and bright-field (middle) images of HEK293T cells expressing EGFP-tagged TRPM3α2, TRPM3α3, TRPM3γ2, or TRPM3γ3. Scale bar: 70 µm. Right panels show magnified fluorescent (upper) and bright-field (lower) images of a single HEK293T cell. Scale bar: 20 µm. (PDF 5262 kb) [file 12576_2019_677_MOESM1_ESM.pdf]

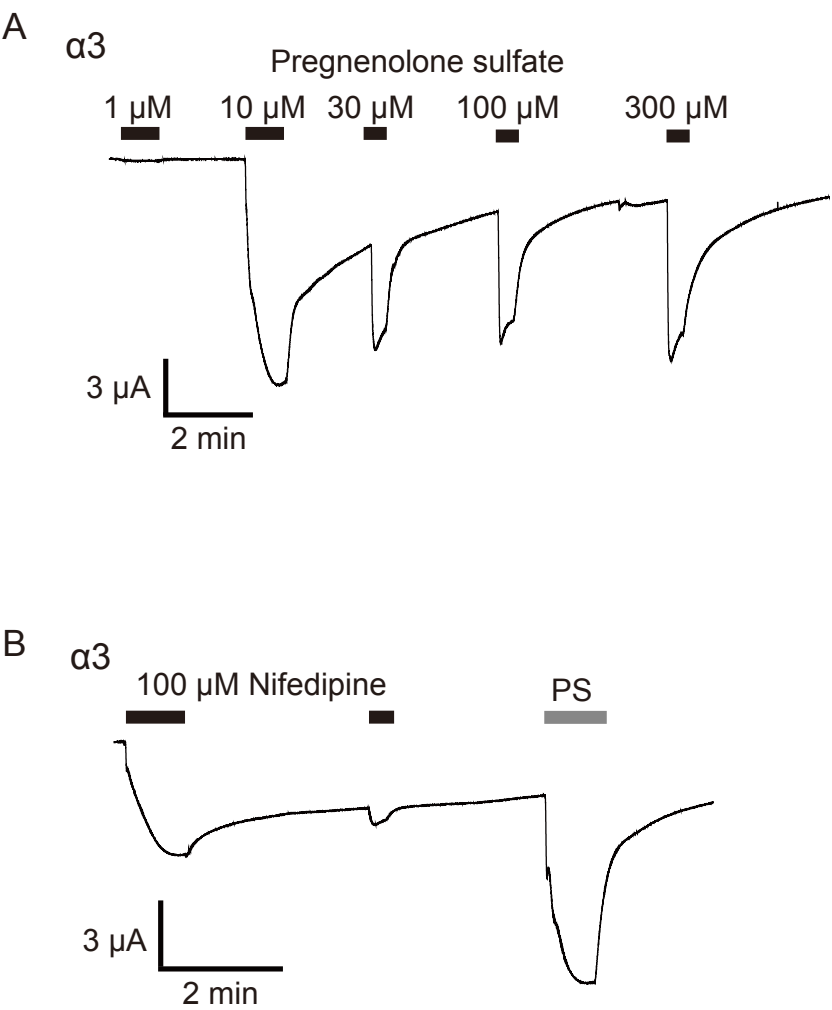

Supplement: Supplementary file 2 — Supplementary Figure 2. (A) Representative trace of TRPM3α3 currents activated by pregnenolone sulfate (1 to 300 µM). (B) Representative trace of TRPM3α3 currents activated by nifedipine (100 µM). Pregnenolone sulfate (PS, 100 µM) was applied after nifedipine. (PDF 841 kb) [file 12576_2019_677_MOESM2_ESM.pdf]

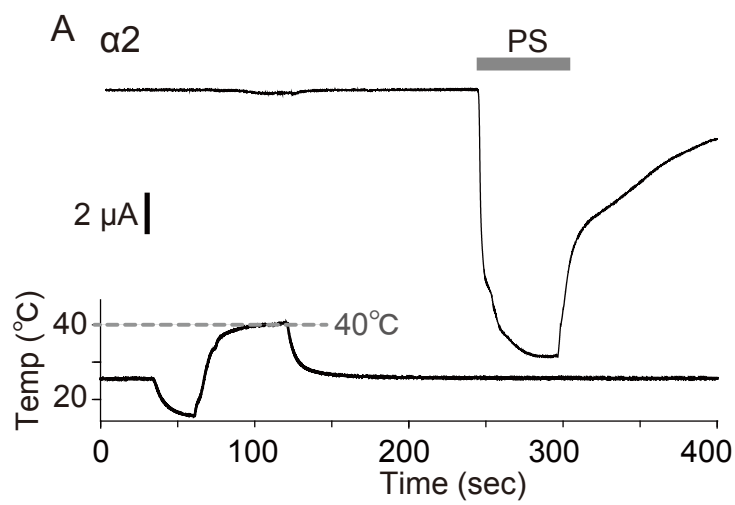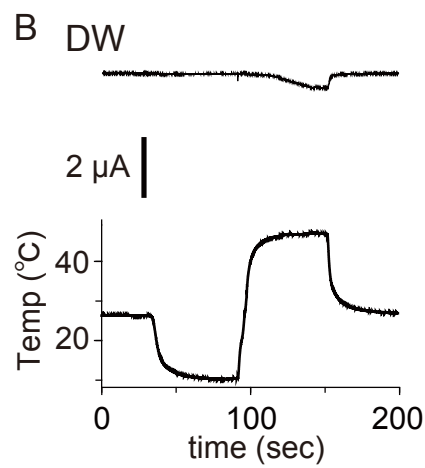

Supplement: Supplementary file 3 — Supplementary Figure 3. (A) Representative trace of TRPM3α2 currents activated by rapid temperature changes up to 40 °C in Xenopus oocytes. Pregnenolone sulfate (PS, 100 µM) was used to confirm TRPM3α2 expression. (B) Representative trace of currents induced by rapid temperature changes up to 46 °C in water-injected Xenopus oocytes. (PDF 521 kb) [file 12576_2019_677_MOESM3_ESM.pdf]
